# In Vitro Cell Viability and Migration Inhibitory Effects of Isorhamnetin in Non-Small Cell Lung Cancer Cells

**DOI:** 10.3390/biomedicines14050951

**Published:** 2026-04-22

**Authors:** Chengjing Shen, Taierpuke Maimaiti, Gulijikere Kuerban, Mireguli Abulimiti, Jialu Hu, Zilala Yalihong, Aikebaier Maimaiti

**Affiliations:** College of Pharmacy, Xinjiang Medical University, Engineering Research Center of Xinjiang and Central Asian Medicine Resources, Ministry of Education, Urumqi 830017, China; scj1234562025@163.com (C.S.);

**Keywords:** isorhamnetin, non-small cell lung cancer, apoptosis, cell cycle, transcriptomics, molecular mechanism

## Abstract

**Background**: Lung cancer remains the leading cause of cancer-related mortality worldwide, with non-small cell lung cancer (NSCLC) accounting for approximately 85% of all cases. Isorhamnetin (ISO), a natural dietary flavonoid, has demonstrated potent anti-lung cancer activity in cell models. However, its precise mechanism of action within the complex landscape of NSCLC remains to be fully elucidated. **Methods**: The effects of ISO on NSCLC cell viability, apoptosis, and cell cycle distribution were assessed in A549 and H1650 cells using the MTT assay, Annexin V-FITC/PI staining, and flow cytometry. Wound healing and Transwell assays were employed to evaluate the isorhamnetin impact on cell migration, invasion, and adhesion. To investigate the underlying molecular mechanisms, RNA sequencing (RNA-seq) was performed, followed by validation of key target genes and proteins using qRT-PCR and Western blot analysis. **Results**: ISO treatment elicited a significant, dose- and time-dependent inhibition of NSCLC cell viability, which coincided with a marked induction of apoptosis. Cell cycle analysis revealed that ISO triggered an S-phase arrest. Transcriptomic profiling identified *ELFN1* and TMEM186 as significantly upregulated genes, while *SETDB1* was downregulated in a concentration-dependent manner; this was accompanied by a concomitant upregulation of *FGFBP1* protein expression. Functionally, ISO effectively suppressed the migratory, invasive, and adhesive capabilities of both cell lines. **Conclusions**: Our findings demonstrate that ISO exerts a potent anti-proliferative and anti-metastatic effect on NSCLC cells. The underlying mechanism is multifaceted, involving the induction of apoptosis and cell cycle arrest, coupled with the modulation of a novel regulatory network centered on *ELFN1*, *TMEM186, SETDB1*, and *FGFBP1*. These results provide new mechanistic insights into the anti-tumor pharmacology of isorhamnetin and highlight its potential as a therapeutic agent targeting both cancer cells and their supporting microenvironments.

## 1. Introduction

Lung cancer is one of the malignant tumors with a globally high morbidity and mortality, and its incidence and death rates are 11.6% and 18.4%, respectively [1]. Lung cancer is divided into two categories: small cell lung cancer (SCLC) and non-small cell lung cancer (NSCLC) [2]. SCLC has three subtypes, namely, SCLC-A, SCLC-N, and SCLC-P, and it accounts for 10–15% of lung cancer cases [3]. NSCLC includes adenocarcinoma, squamous cell carcinoma and large cell carcinoma, which account for 85–90% of lung cancer cases [4]. Although early-stage NSCLC patients can be cured by surgical resection [1], most patients are diagnosed at middle or advanced stages [5] and eventually develop tumor metastasis. Moreover, targeted therapies and immunotherapies have significantly improved NSCLC patient outcomes in recent years, but the five-year survival rate of metastatic NSCLC patients is still less than 5% as resistance develops in most patients receiving current therapies [6]. Hence, there is growing interest in exploring new therapies and new anti-NSCLC drugs with clear molecular mechanisms [7,8].

More and more studies have documented that traditional Chinese medicine plays a unique role in cancer prevention and treatment. Natural Chinese medicines not only exhibit potent anti-tumor effects in various cancers but also reduce the drug resistance and toxicity of different therapies [9]. Isorhamnetin is a natural flavonoid found in several plants, including sea buckthorn and Ginkgo biloba [10]. Pharmacological studies have demonstrated that isorhamnetin possesses antitumor [11], anti-inflammatory, antiviral, and antioxidant activities [12]. Many articles have reported that isorhamnetin exhibits strong anti-lung cancer effects in vitro and in vivo. For example, Lu et al. [13]. demonstrated that isorhamnetin inhibits A549 cell invasion and migration via the Akt/ERK signal pathway. It is reported that isorhamnetin enhanced A549 cell radiosensitivity by mediating the IL-13 and NF-κB signaling pathways [14]. Li et al. [15] found that isorhamnetin inhibited A549 cell growth in vitro with the IC50 44.45 ± 6.21 μg/mL, and suppressed tumor growth in vivo. It also upregulated Bax, Caspase-3 and P53 protein expression levels, and downregulated Bcl-2, cyclinD1 and PCNA protein expression levels in vitro and in vivo. Zhang et al. [16] have documented that isorhamnetin inhibits A549 cell proliferation and apoptosis. Moreover, Ruan et al. [17] have reported that isorhamnetin inhibits A549 cell proliferation and induces apoptosis via regulation of the mitochondrial autophagy pathway. Although the anti-lung cancer potential of isorhamnetin has been reported, its mechanisms in NSCLC remain unclear. While previous studies have established the anti-proliferative and pro-apoptotic effects of isorhamnetin in NSCLC [13,14,15,16,17], its complete molecular mechanism remains incompletely understood, and its direct protein targets have not been systematically identified. The present study addresses this gap and advances the field by (1) transcriptomic screen (RNA-seq) to identify novel responsive genes and by (2) experimentally validating, for the first time, that isorhamnetin concurrently modulates the expression of four key proteins—*ELFN1*, *TMEM186*, *SETDB1*, and *FGFBP1*—linking them to its anti-NSCLC phenotypes. Unlike prior research focusing on single pathways or phenotypic descriptions, our work integrates multi-target discovery with functional validation to propose a novel, multi-faceted mechanism of action, providing a deeper and more systematic understanding of isorhamnetin’s therapeutic potential against NSCLC.

A549 and H1650 are commonly used NSCLC cell lines and are widely applied in in vitro and in vivo experiments for drug development and mechanism exploration of anti-cancer agents in lung cancer research. A549 is a widely used NSCLC model for proliferation/migration and drug screening due to its stable epithelial-like morphology and ease of culture [18,19]. The H1650 cells also have a unique gene mutation profile that makes them the preferred model for targeted therapy research [20,21], and this cell line has shown significant advantages in mimicking tumor microenvironment interactions and signaling pathway regulation. Therefore, in this article, we studied the in vitro cell viability and cell migration inhibitory effects of isorhamnetin using A549 and H1650 cell lines, and the possible molecular mechanism involved.

## 2. Materials and Methods

### 2.1. Materials

Isorhamnetin was bought from Aladdin Biochemical Technology (Shanghai, China; purity ≥ 98%). The F-12K medium, RPMI 1640 medium, fetal bovine serum (FBS), collagenase I, 3-[4,5-dimethyl-2-thiazolyl]-2,5-diphenyl-2-tetrazoliumbromide (MTT), penicillin and streptomycin, 0.25% trypsin-EDTA, dimethyl sulfoxide (DMSO) and Trizol reagent were procured from Invitrogen (Grand Island, NY, USA). Prime Script RT reagent Kit with gDNA Eraser and TB Green^®^ Premix Ex Taq TM with ROX were procured from Takara Bio (Tokyo, Japan). The primer was purchased from Qingke Bio (Beijing, China). The apoptosis detection kit (annexin V-FITC) was obtained from Biomiga (San Diego, CA, USA). Nuclease-free water was purchased from Biosharp (Beijing, China). Antibodies against ELFN1 were purchased from Biorbyt (Cambridge, UK). Antibodies against TMEM186 and β-actin were purchased from Affinity Biosciences (liyang, Jiangsu, China). Antibodies against SETDB1, FOSB, H3K9me3, FGFBP1, NPTX1, MAP2K6, EGR, and GBP4, as well as Goat Anti-Rabbit IgG and Goat Anti-Mouse IgG, were obtained from Abcam (Cambridge, UK).

### 2.2. Cell Culture

The A549 (TCHu150) and H1650 (CBP60084) NSCLC cells were kindly supplied by the Cell Bank of Type Culture Collection (Chinese Academy of Sciences, Shanghai, China). FBS (10% heat-inactivated) and 1% penicillin–streptomycin was added to F-12K and RPMI 1640 medium, respectively. These were then incubated at 37 °C in a humidified environment with 5% CO_2_. After dissolving isorhamnetin in DMSO to a final concentration of 0.1%, the cells were exposed to several concentrations of isorhamnetin.

### 2.3. Cell Viability Assay

The vitality of the A549 and H1650 cells was assessed using MTT reagent (Cat. No. M7007, Abmole Bioscience, Houston, TX, USA) in accordance with Maimaiti et al. Isorhamnetin was applied to A549 and H1650 cells with several concentrations (0–200 μM). Then, the MTT assay was used to measure the of cell viability of A549 and H1650 cells at different times (24 h, 48 h, and 72 h) following the manufacturer’s instruction [22].

### 2.4. Apoptosis Analysis

A549 and H1650 cells were cultured and treated with isorhamnetin (0–200 μM) for 24 h. The same procedure was conducted as previously described in article [22], and an Annexin V-FITC staining kit (Cat. No. 556547) was used for the detection of Annexin V binding. A BD LSRII flow cytometer (Becton, Dickinson and Company, Franklin Lakes, NJ, USA) was then used to analyze the samples. three independent experiments were performed, with *n* = 3 wells per group in each experiment

### 2.5. Cell Cycle Analysis

A549 and H1650 cells were cultured and treated with isorhamnetin (0–200 μM) for 24 h. The determination of cell cycle distribution was done using the same procedure as previously mentioned in the article, and using a cell cycle detection kit (Cat. No. 550825). A BD LSRII flow cytometer (BD, Franklin Lakes, NJ, USA) was then used to analyze each sample. Three independent experiments were performed, with *n* = 3 wells per group in each experiment. The obtained data were analyzed using one-way analysis of variance (ANOVA) in GraphPad Prism (9.5 version). Tukey’s multiple comparisons test was used to compare the means between groups. Result were considered significant at *p* < 0.05.

### 2.6. Wound Healing Assay

A549 and H1650 cells were seeded at a density of 2 × 10^5^ cells per well in a six-well plate. The next day, three scratch wounds were created on each dish using a 200 µL pipette tip through the confluent cell monolayer. Then, isorhamnetin was applied to the cells for 0, 12, and 24 h. Images were taken using a phase contrast microscope (DMI 8, Leica Microsystems, Wiesler, Germany).

### 2.7. Transwell Assay

Transwell chambers (8.0 μm; Beijing Labgic Tech, Beijing, China) were used for assessment of A549 and H1650 cell movement. The inserts were pre-coated with Matrigel by adding 50 μL of ice-cold Matrigel solution (diluted 1:8 in serum-free medium) to the upper chamber, followed by incubation at 37 °C for 1 h to allow gelation. Briefly, the upper chamber was filled with A549 and H1650 cells (4 × 10^4^ cells/well) in serum-free media, while the bottom chamber was filled with 600 µL of culture medium containing 15% FBS. After that, isorhamnetin was used to treat the cells for 24 h. The following day, 4% formaldehyde and 0.1% crystal violet were used to fix and stain the invasive cells. Images were taken using a phase contrast microscope (DMI 8, Leica Microsystems). The invasion test was conducted in the same manner as the above procedure, except that the Matrigel (Corning, Corning, NY, USA) was used to precoat the Transwell chambers.

### 2.8. Adhesion Assay

A549 and H1650 cells were seeded in the six-well plates and treated with isorhamnetin (0–200 μM) for 24 h. Then, the collected cells (20,000 cells per well) were cultured in 96-well plates that were covered with Matrigel. For the 96-well plate-coating procedure, 50 µL of Matrigel solution (diluted 1:15 in serum-free medium) was added to each well. The plate was then incubated at 37 °C overnight to allow for gel polymerization. Following incubation, the gel was washed twice with phosphate-buffered saline (PBS). Subsequently, 100 µL of 3% bovine serum albumin (BSA) solution was added to each well and incubated for 2 h at 37 °C to block nonspecific binding sites. Finally, the wells were washed twice with PBS to make ready for use, before being incubated for 1 h. After the incubation period, unattached cells were removed by gently aspirating the medium from each well, followed by washing the wells twice with PBS (phosphate-buffered saline). After the nonadherent cells were cleaned, the fresh media (100 μL) and MTT (10 μL) were added to each well. Then, the sample’s optical density under a wavelength of 570 nm was measured using a microplate reader (Thermo Fisher Scientific Inc., Waltham, MA, USA). Three independent experiments were performed, with *n* = 5 wells per group in each experiment.

### 2.9. RNA-Seq and Pathway Enrichment Analysis

TRIzol^®^ reagent was used for extracting the total RNA according to the manufacturer’s recommendation. The Agilent 2100 Bioanalyzer (Santa Clara, CA, USA) (GBSF) with the Agilent High Sensitivity DNA kit was used to determine the quality of total RNA. The libraries for RNA-seq were prepared using the MGIEasy RNA Lab Chip kit and the MGIEasy DNA a-dapters-96 (plate) kit. MGIEasy magnetic beads and the MGIEasy Cyclization Module Kit were sequenced using the MGI-SEQ-2000RS high-throughput sequencing reagent kit to obtain 2 × 150 bp paired-end reads. The raw reads were trimmed by removing all adaptor sequences, low quality reads (Q < 30) and short reads (sequence length < 50 bp) to obtain clean reads. The clean reads were compared with the reference genome and obtained alignments by HISAT2 v2.0.4 with default parameters. Reference-based assembly of transcripts and prediction of expression were performed using String Tie. Bioconductor edgeR was used to conduct the ranking of genes based on the differential expression degree. Significantly differentially expressed genes with the following criteria as *p* ≤ 0.05 cut-off values and log2 fold change ≥ |2| in the mean expression were selected for further experiment. Each gene was normalized by using the fragments per kilobase per million mapped reads FPKM method. To capture a wider range of potentially subtle but consistently expressed changes, the initial GO and KEGG enrichment analyses were performed using genes with a *p*-value < 0.05, irrespective of their fold change. For the selection of individual candidates for validation, a combined strategy of statistical significance (*p* < 0.01, |log2FC| > 2), biological plausibility, and manual annotation was employed. The pathway enrichment analysis, including the Ontology Biological Process (GOBP) and Kyoto Encyclopedia of Genes and Genomes (KEGG), was conducted using Metascape online platform (https://metascape.org/gp/, accessed on 12 December 2025) [22].

### 2.10. Real-Time Quantitative Polymerase Chain Reaction (RT-qPCR)

The RT-qPCR experiment was conducted according to Maimaiti et al. [22], with slight modification. The total RNA was extracted using Trizol reagent (Lot no. 15596026; Invitrogen, USA). The concentration and quality of the extracted RNA were determined by Nanodrop 2000c (Thermo Fisher Scientific). Then, the Takara kit and Prime Script™ RT Master Mix reagent (Takara, Tokyo, Japan) were used for cDNA synthesis according to the manufacturer’s protocol. Specific primers for *ELFN1*, *TMEM186*, and *GAPDH* were designed and synthesized, and their sequences are listed in Table 1. Afterwards, Afterwards, the gene expression was measured using the QuantStudio^TM^ 1 Plus Real-Time PCR System (Thermo Fisher Scientific Inc, Waltham, MA, USA) following the following protocol: first step, the temperature was set to 95 °C for 30 s; second step, the temperature was set to 95 °C for 5 s; finally, the temperature was set to 60 °C for 34 s. The first and second steps were run in 40 cycles and the third step was melting. *ELFN1* and *TMEM186* mRNA expressions were measured by recording the intensity of fluorescent emission from cDNA amplification. GAPDH was used as an internal control for mRNA. Finally, we analyzed the data using the 2^−ΔΔCt^ formula.

### 2.11. Western Blot Analysis

With a minor adjustment, Western blot analysis was performed in compliance with Maimaiti et al. [22]. In short, A549 and H1650 cells were centrifuged for 20 min at 13,000× *g*/4 °C after being lysed for 20 min with RIPA. The BCA protein assay kit (Solarbio, Beijing, China) was used for quantification of the protein concentration. Protein samples were subjected to SDS-PAGE using 10% polyacrylamide gels. Then, the proteins were moved to a polyvinylidene difluoride membrane (Millipore Corp. Billerica, MA, USA) and a blocking solution was used to block the protein-blotted membrane for 2 h. After adding antibodies to the blocking buffer at a dilution ratio of 1:1000 or 1:10,000, the membrane was incubated at 4 °C for the entire night. Each membrane was washed three times for 8 min using TBST and incubated for 1 h with either secondary Goat anti-Rabbit IgG (1:10,000) or secondary Goat anti-Mouse IgG (1:10,000). Then, the membrane was washed three times again with TBST for 8 min and detected using an ECL Western blot detection system.

### 2.12. Computational Biology Analysis

Differentially expressed genes (DEGs) from RNA-seq (|log2FC| > 2, adj. *p* < 0.05) were uploaded to the CLUE platform (clue.io) to perform CMap analysis. Connectivity scores and statistical significance (*p*-values) were calculated to identify potential upstream regulators and prioritize high-confidence targets.

The three-dimensional structures of the target proteins (PPARG: 6TSG, NR3C2: 4X9W, DRD2: 6CM4) were retrieved from the Protein Data Bank (PDB). Molecular docking was performed using the CB-Dock2 online server (https://cadd.labshare.cn/cb-dock2/ (accessed on 7 April 2026)).

According to the server protocol, the PDB files were prepared by removing water molecules and adding polar hydrogen atoms. CB-Dock2 automatically identifies the optimal binding pocket via a cavity detection algorithm and performs blind docking. The top-ranked poses were selected based on the docking score (Vina score, kcal/mol) and cluster size. Key interacting residues and binding free energies were analyzed to evaluate the binding stability of isorhamnetin.

### 2.13. Statistical Analysis

The data were analyzed by GraphPad Prism 9.8. One-way ANOVA was used to analyze the significance of different groups, including Dunnett’s test for single group comparison, and Tukey’s HSD test for multiple group comparison. All experiments were performed at least three times. The *p*-value < 0.05 was considered statistically significant.

## 3. Results

### 3.1. Isorhamnetin Inhibited NSCLC Cell Viability

**Figure 1:**
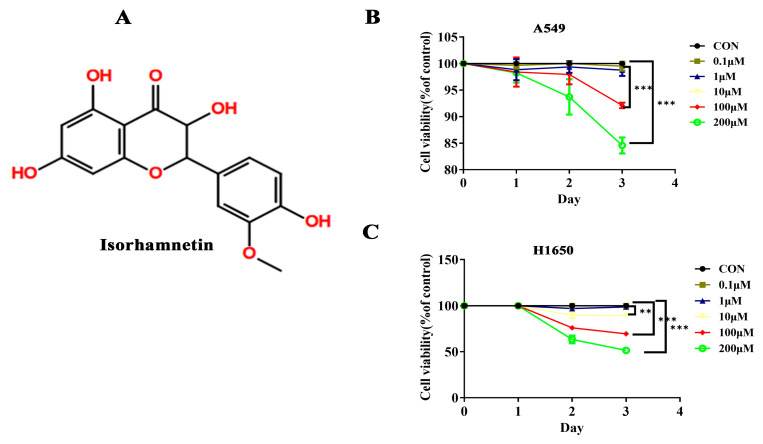
Isorhamnetin inhibited NSCLC cell viability. (**A**) Chemical structure of isorhamnetin. (**B**) and (**C**) show the respective viabilities of A549 and H1650 cells treated with isorhamnetin (0.1–200 μM) for 24, 48, and 72 h. One-way ANOVA was used to determine the percentage of cell viability. All experiments were conducted three times (*n* = 3, mean ± SD), and significance was considered at ** *p* < 0.01, *** *p* < 0.01.

The effects of isorhamnetin on the viability of A549 and H1650 cells were determined. The MTT results show that isorhamnetin treatment (Figure 1B,C) time- and concentration-dependently reduced the viability of A549 and H1650 cells. The results suggest that isorhamnetin inhibited A549 and H1650 cell viability at the moderate or low level. Again, it is clear from Figure 1B,C that isorhamnetin treatment decreased the viability of H1650 cells more than that of A549 cells. These results indicate that the inhibitory effects of isorhamnetin vary with different types of NSCLC cells.

### 3.2. Isorhamnetin Induced NSCLC Cell Apoptosis and Cell Cycle Arrest

**Figure 2:**
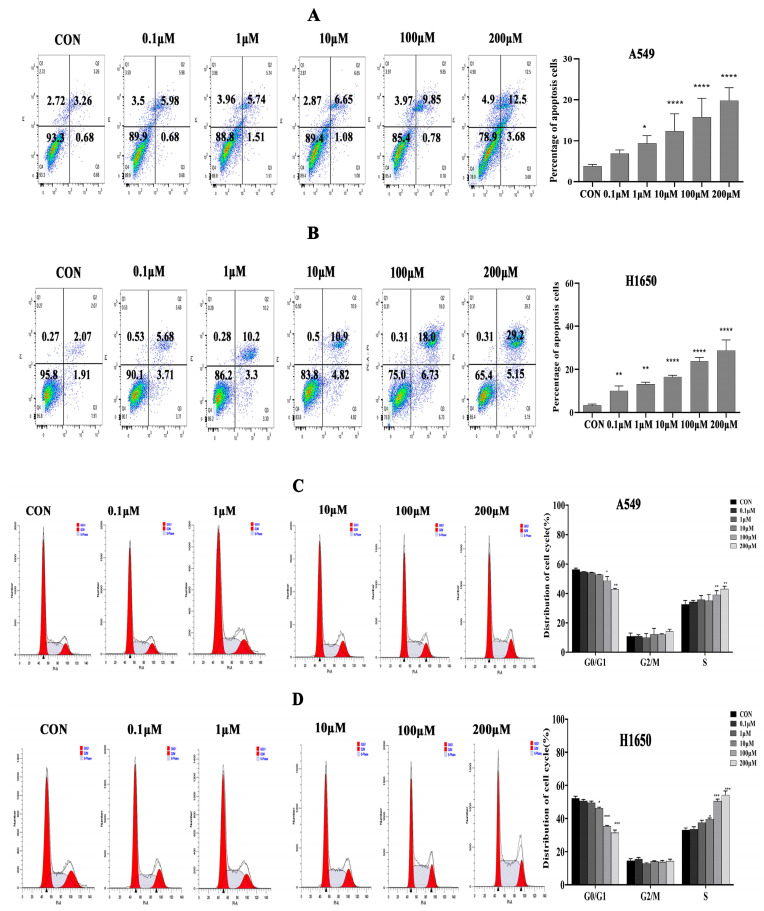
Isorhamnetin induced apoptosis and cell cycle arrest in the S phase. (**A**,**B**) Effect of isorhamnetin (0.1–200 μM) on the cell apoptosis of A549 and H1650 cells, respectively, at 24 h. (**C**,**D**) Flow cytometry was performed to detect the effect of isorhamnetin (0.1–200 μM) on the cycle of respective A549 and H1650 cell lines. One-way ANOVA was used to determine the percentage of apoptosis and cell cycle arrest. All experiments were conducted three times (*n* = 3, mean ± SD), and significance was considered at * *p* < 0.05, ** *p* < 0.01, *** *p* < 0.001, and **** *p* < 0.0001.

To elucidate the mechanism of isorhamnetin on the NSCLC cell viability inhibitory effect, the apoptosis and cell cycle phase distribution of A549 and H1650 cells were then measured after treatment with isorhamnetin. The results showed that isorhamnetin treatment concentration-dependently increased Annexin V-stained A549 and H1650 (*p* < 0.01) NSCLC cells at 24 h (Figure 2A,B). Moreover, compared with the control, the cell cycle S phase was significantly increased after treatment with isorhamnetin for 24 h, suggesting that isorhamnetin may inhibit NSCLC cell viability via induction of apoptosis and cell cycle S phase arrest (*p* < 0.01) (Figure 2C,D).

### 3.3. Isorhamnetin Inhibited NSCLC Cell Invasion, Migration and Adhesion

**Figure 3:**
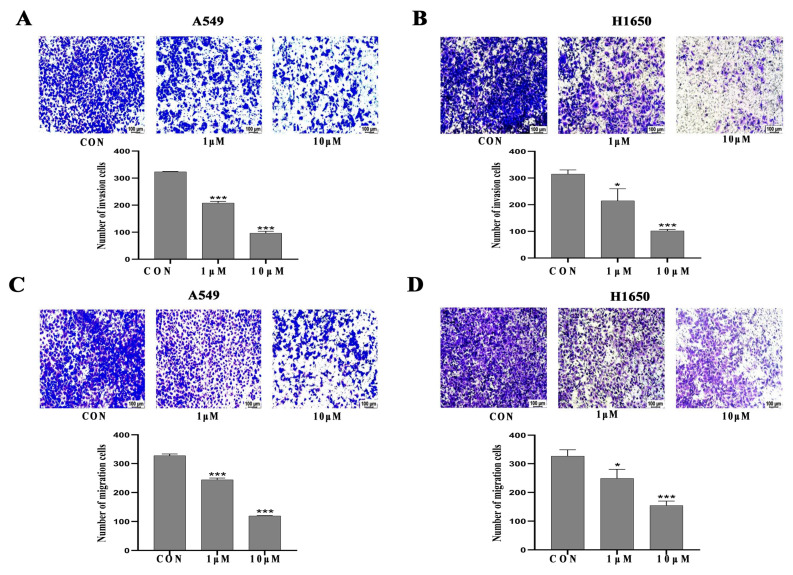
Isorhamnetin inhibited NSCLC cell invasion and migration. (**A**,**B**) Transwell invasion assay was applied to analyze the effect of isorhamnetin (0.1–10 μM) on the invasion of A549 and H1650 cells. (**C**,**D**) Transwell migration assay was applied to analyze isorhamnetin (0.1–10 μM) on the invasion of A549 and H1650 cells. One-way ANOVA was used to determine the percentage of invasive and migratory cells. All experiments were conducted three times (*n* = 3, mean ± SD), and significance was considered at * *p* < 0.05 and *** *p* < 0.01.

**Figure 4:**
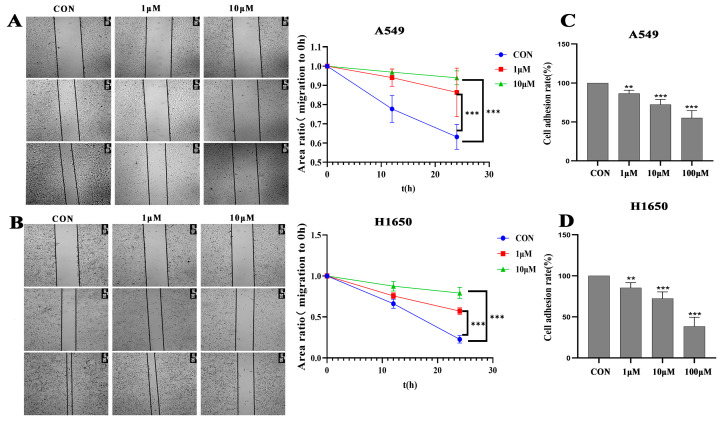
Isorhamnetin inhibited NSCLC cell wound healing and adhesion. (**A**,**B**) Wound healing was applied to analyses of isorhamnetin (0.1–10 μM) on the migration of A549 and H1650 cells. The wound recovery values of each sample were quantified by comparing with 0 h points. (**C**,**D**) A549 and H1650 cells treated with 0.1–10 μM isorhamnetin for 24 h. One-way ANOVA was used to determine the percentage of adhesion and wound healing cells. All experiments were conducted three times (*n* = 3, mean ± SD), and significance was considered atand ** *p* < 0.01, *** *p* < 0.01.

Transwell invasion, wound healing, Transwell migration and adhesion assays were conducted to examine the effects of isorhamnetin on NSCLC cell invasion, migration and adhesion. The results showed that isorhamnetin strongly inhibited the invasion of A549 and H1650 (*p* < 0.01) NSCLC cells in a concentration-dependent manner (Figure 3A,B). The wound healing assay results demonstrated that isorhamnetin (Figure 3C,D) remarkably reduced the migration of A549 and H1650 (*p* < 0.01) NSCLC cells in a concentration- and time-dependent manner. The Transwell migration assays further confirmed that isorhamnetin treatment concentration-dependently inhibited the A549 and H1650 (*p* < 0.01) NSCLC cell migration compared with the control (Figure 4A,B). In addition, a significant concentration-dependent reduction in A549 and H1650 (*p* < 0.01) NSCLC cell adhesion was observed after treatment with isorhamnetin (Figure 4C,D).

### 3.4. RNA-Seq Transcriptome and Pathway Enrichment Analysis

**Figure 5:**
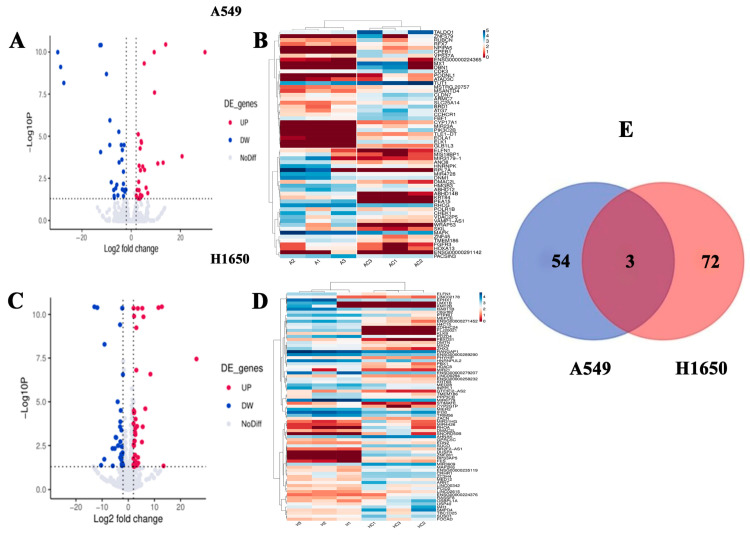
Gene expression profile of isorhamnetin-treated NSCLC cells by RNA-seq analysis. (**A**) The volcano map of isorhamnetin-treated or untreated A549 cells. (**B**) The heat map of isorhamnetin-treated or untreated A549 cells. (**C**) The volcano map of isorhamnetin-treated or untreated H1650 cells. (**D**) The heat map of isorhamnetin-treated or untreated H1650 cells. (**E**) Venn diagram of significantly differentially expressed genes of isorhamnetin-treated A549 and H1650 cells.

**Figure 6:**
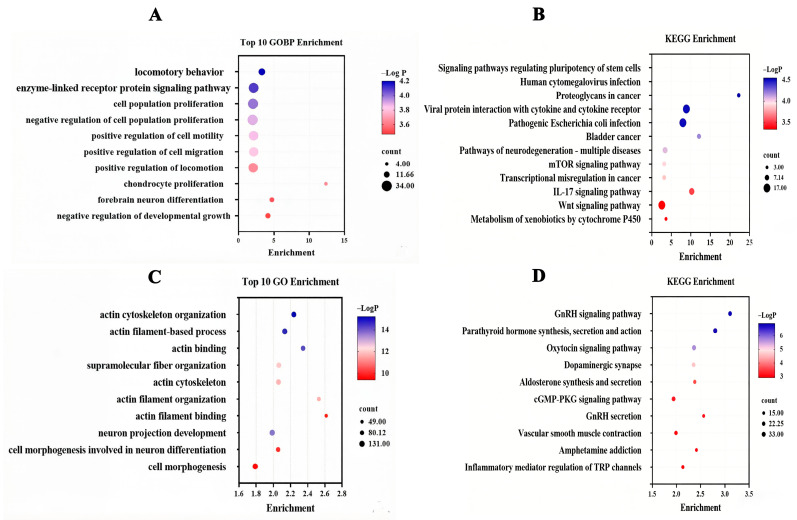
Gene terms and KEGG pathways enriched by differentially expressed genes inA549 and H1650 cells. (**A**,**C**) The ten top category terms of GO analysis (A549and H1650 cells). The y-axes correspond to the GO terms, and the x-axes show the enrichment. (**B**,**D**) The signaling pathways of KEGG analysis (A549 cell). The y-axes correspond to the pathways and the X-axes show the enrichment.

To evaluate the mechanism of the cell viability and cell migration inhibitory effects of isorhamnetin in NSCLC, the effect of isorhamnetin treatment (10 μM, 24 h) on the A549 and H1650 cells’ gene expression profiles was determined. RNA-seq transcriptome analysis demonstrated that 826 genes (*p* < 0.05) were significantly differentially expressed, including 359 genes that were upregulated and 467 genes that were downregulated in isorhamnetin-treated A549 cells (Tables S1 and S2). GO analysis showed that 184 gene ontology categories were found to be significantly enriched by significantly differentially expressed genes (SDGs, *p* < 0.05). Among these, SDGs were mainly involved in locomotory behavior, the enzyme-linked receptor protein signaling pathway, cell population proliferation, negative regulation of cell population proliferation, positive regulation of cell motility, positive regulation of cell migration, positive regulation of locomotion, chondrocyte proliferation, forebrain neuron differentiation, and negative regulation of developmental growth (Figure 5A and Table S4). KEGG analysis demonstrated that 12 signal pathways were significantly enriched by SDG (*p* < 0.05). Among these, signaling pathways regulating pluripotency of stem cells, human cytomegalovirus infection, proteoglycans in cancer, viral protein interaction with cytokine and cytokine receptor, pathogenic Escherichia coli infection, bladder cancer, pathways of neurodegeneration-multiple diseases, mTOR signaling pathway, transcriptional misregulation in cancer, IL-17 signaling pathway, Wnt signaling pathway, and metabolism of xenobiotics by cytochrome P450 were mainly involved (Figure 6B and Table S5). Moreover, 3326 genes (*p* < 0.05) were significantly differentially expressed including 1632 genes were upregulated, and 1694 genes were downregulated in isorhamnetin-treated H1650 cells (Tables S6 and S7). GO analysis showed that 1194 gene ontology categories were found to be significantly enriched by SDG, (*p* < 0.05). Among these, SDG were mainly involved in actin cytoskeleton organization, actin filament-based process, actin binding, supramolecular fiber organization, actin cytoskeleton, actin filament organization, actin filament binding, neuron projection development, cell morphogenesis involved in neuron differentiation, and cell morphogenesis (Figure 6C and Table S9). KEGG analysis demonstrated that 82 signal pathways were significantly enriched by SDG (*p* < 0.05). Among these, the GnRH signaling pathway, parathyroid hormone synthesis, secretion and action, oxytocin signaling pathway, dopaminergic synapse, aldosterone synthesis and secretion, cGMP-PKG signaling pathway, GnRH secretion, vascular smooth muscle contraction, amphetamine addiction, and inflammatory mediator regulation of TRP channels were mainly involved (Figure 6D and Table S10). As shown in Figure 5A–E and (Tables S3 and S8), *ELFN1* and *TMEM186* (>2-fold change) genes consistently upregulated in both A549 and H1650 cells after treatment of isorhamnetin (Figure 5E) and Table S8. However, the *DMAC2L* gene was upregulated in isorhamnetin-treated A549 cells, while downregulated in isorhamnetin-treated H1650 cells. Although the global GO/KEGG enrichment based on the p-value threshold did not specifically highlight pathways, we identified two genes—*ELFN1* (encoding a synaptic protein) and *TMEM186* (encoding a putative mitochondrial membrane protein)—as candidates of interest due to their consistent differential expression and uncharacterized roles. To gain deeper insights into the molecular mechanism, we performed PPI network analysis. Specifically, *ELFN1* is associated with receptor signaling pathways (e.g., *FGFR3*), while TMEM186 is linked to intracellular trafficking and mitochondrial function (e.g., *DNM1*). We interpret this as evidence of a ‘Convergent Mechanism’ rather than a direct interaction. Isorhamnetin appears to simultaneously disrupt extracellular signal transduction (via *ELFN1*) and intracellular metabolic homeostasis (via *TMEM186*). This dual targeting of independent pathways likely contributes to the compound’s potent efficacy, as it attacks the cancer cells from two different fronts. Intriguingly, functional enrichment analysis revealed terms seemingly unrelated to lung cancer, such as “forebrain neuron differentiation” and “Human cytomegalovirus infection”. We interpret the former as evidence of isorhamnetin’s impact on developmental signaling pathways (e.g., Wnt/Notch) that regulate cancer stemness. The latter likely reflects significant dysregulation of cytokine–cytokine receptor interactions, indicating a potential immunomodulatory effect. These findings collectively suggest a multi-targeted mechanism of action.

### 3.5. Effects of Isorhamnetin on the ELFN1 and TMEM186 Gene and Protein Expression Levels in NSCLC Cells

**Figure 7:**
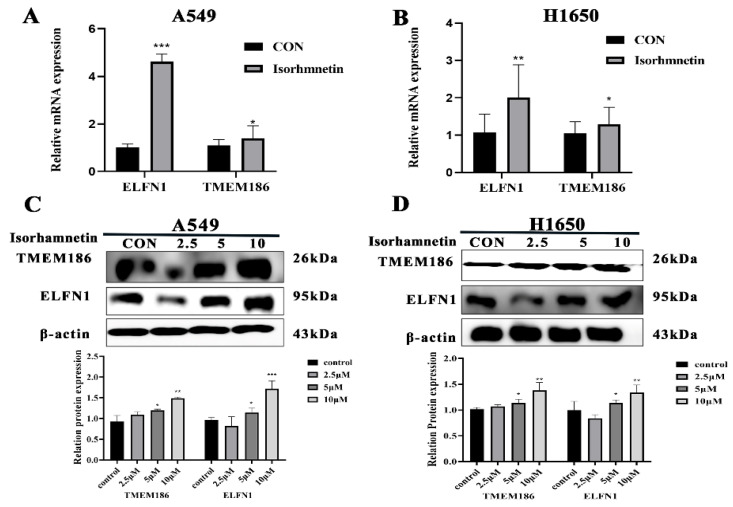
Isorhamnetin upregulated *ELFN1* and *TMEM186* gene and protein expression levels in NSCLC cells. (**A**,**B**) Effect of isorhamnetin on the respective gene expression levels of *ELFN1* and *TMEM186*. (**C**,**D**) Effect of isorhamnetin on the protein expression levels of *ELFN1* and *TMEM186*. The percentage of protein expression of each sample was determined using one-way ANOVA. All experiments were conducted three times (*n* = 3, mean ± SD), and significance was considered at * *p* < 0.05, ** *p* < 0.01 and *** *p* < 0.01.

To confirm the RNA-seq results, Q-PCR and Western blot experiments were conducted. As shown in (Figure 7A,B), compared with the control, *ELFN1* (*p* < 0.01) and *TMEM186* (*p* < 0.05) genes were significantly upregulated in isorhamnetin-treated A549 and H1650 cells in a concentration-dependent manner. Moreover, Western blot results showed that isorhamnetin treatment upregulated *ELFN1* (*p* < 0.01) and *TMEM186* (*p* < 0.01) protein expression levels in A549 and H1650 cells compared with the control (Figure 7C,D).

### 3.6. Effects of Isorhamnetin on H3K9me3, FosB, SETDB1, FGFBP1, NPTX1, MAP2K6, EGR1, EGR and GBP1-5protein Expression Levels in NSCLC Cells

**Figure 8:**
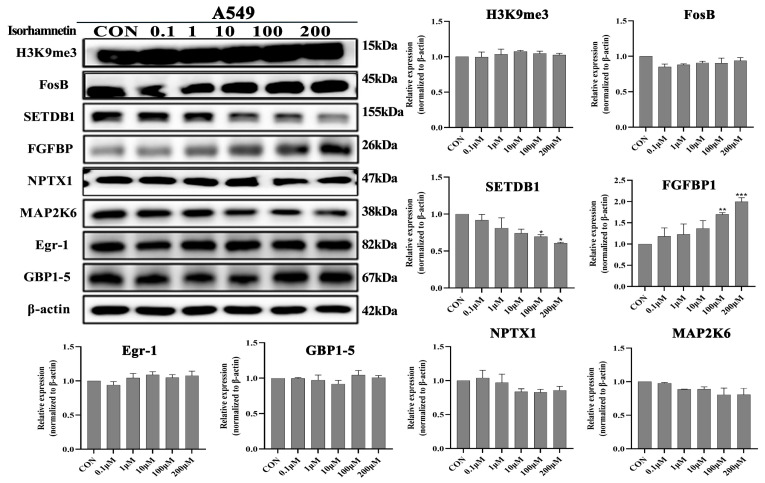
Isorhamnetin upregulated FGFBP1 protein expression level, and downregulated SETDB1 protein expression level in A549 cells. Concentration effects of isorhamnetin on the protein expression levels of H3K9me3, FosB, SETDB1, FGFBP1, NPTX1, MAP2K6, EGR1 and GBP4. The percentage of protein expression of each sample was determined using one-way ANOVA. All experiments were conducted three times (*n* = 3, mean ± SD) and significance was considered at * *p* < 0.05, ** *p* < 0.01 and *** *p* < 0.01.

**Figure 9:**
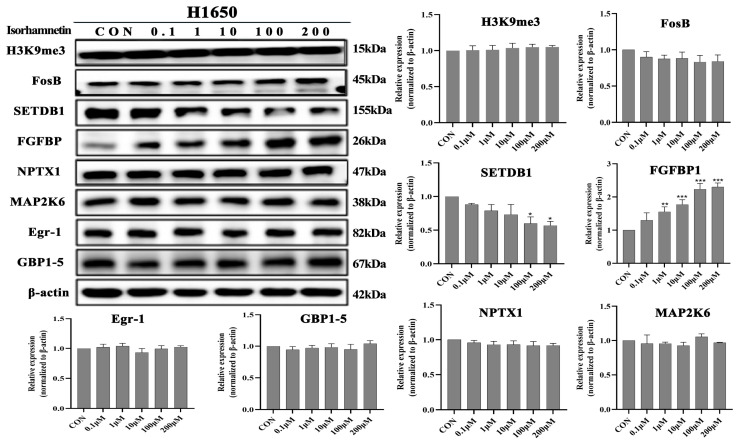
Isorhamnetin upregulated *FGFBP1* protein expression level, and downregulated *SETDB1* protein expression level in H1650cells. Concentration effects of isorhamnetin on the protein expression levels of *H3K9me3*, *FosB*, *SETDB1*, FGFBP1, *NPTX1*, *MAP2K6*, *EGR1* and *GBP4*. The percentage of protein expression of each sample was determined using one-way ANOVA. All experiments were conducted three times (*n* = 3, mean ± SD), and significance was considered at * *p* < 0.05, ** *p* < 0.01 and *** *p* < 0.01.

Our previous study has showed that the water extract of *Cuscuta chinensis* Lam upregulated *FGFBP1*, *FOSB* and *NPTX1* gene expression levels and downregulated *EGR1*, *GBP4* and *MAP2K6* gene expression levels in vitro and in vivo [22]. Moreover, SETDB1 reversibly catalyzes *H3K9* and negatively regulates *FOSB* expression. It is reported that isorhamnetin rich in *Cuscuta chinensis* Lam, and one of the active compounds of it. Therefore, we also investigated the regulatory effect of isorhamnetin on the above proteins. The Western blot results showed that isorhamnetin treatment concentration-dependently upregulated *FGFBP1* (*p <* 0.01) protein expression levels and downregulated *SETDB1* (*p* < 0.05) protein expression levels in A549 and H1650 lung cancer cells (Figure 8). However, isorhamnetin treatment did not affect the protein expression levels of *H3K9me3*, *FOSB*, *NPTX1*, *MAP2K6*, *EGR1* and *GBP1* in both cells (Figure 9).

### 3.7. Identification of Potential Targets via CMap and Molecular Docking

To further elucidate the multi-target mechanism of isorhamnetin, we performed a CMap analysis to identify potential molecular targets associated with the transcriptomic signature of isorhamnetin treatment. By querying the L1000 dataset with differentially expressed genes (|log2FC| > 2, adj. *p* < 0.05), we identified PPARG and NR3C2 as high-confidence candidates, exhibiting strong positive (concordance = 0.511, *p* = 0.001) and negative (concordance = −0.431, *p* = 0.007) connectivity scores, respectively. Additionally, DRD2 was significantly enriched among the top-ranked perturbagens (concordance = −0.390, *p* = 0.016).

**Figure 10:**
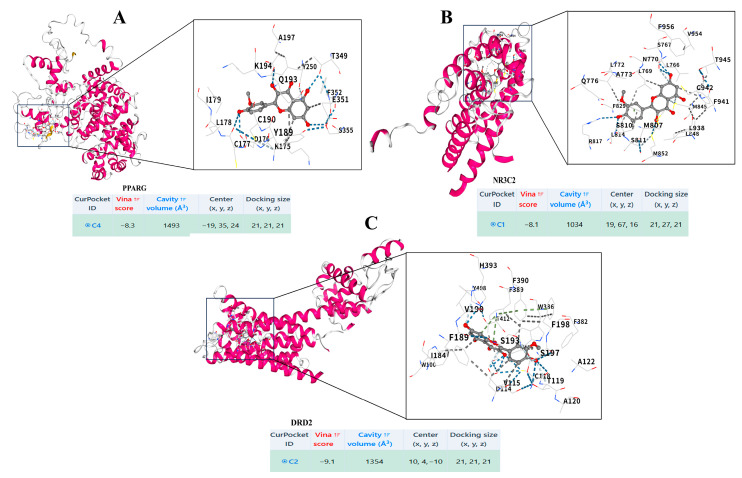
Molecular docking of isorhamnetin with PPARG, NR3C2, and DRD2. (**A**) Isorhamnetin docked into PPARG (inset: binding pocket with key residues; table: Vina score = −8.3, cavity volume = 1493 Å^3^). (**B**) Isorhamnetin bound to NR3C2 (inset: interacting residues; table: Vina score = −8.1, volume = 1034 Å^3^). (**C**) Isorhamnetin docked into DRD2 (inset: ligand-binding pocket; table: Vina score = −9.1, volume = 1354 Å^3^). Dashed lines represent hydrogen bonds/hydrophobic interactions; pocket parameters (ID, score, volume, center, size) are listed below each subfigure.

To further validate the physical plausibility of these predictions, we performed molecular docking simulations. As shown in Figure 10, isorhamnetin formed stable complexes with all three targets, displaying favorable binding energies (Vina scores: PPARG: −8.3 kcal/mol; NR3C2: −8.1 kcal/mol; DRD2: −9.1 kcal/mol). Detailed interaction analysis revealed that isorhamnetin engaged in key hydrogen bonding and extensive hydrophobic contacts within the ligand-binding pockets. These computational findings confirm the multi-target nature of isorhamnetin, thereby broadening the mechanistic landscape of its anti-tumor activity.

## 4. Discussion

Isorhamnetin, a bioactive natural flavonoid, has been reported to exert potent anti-lung cancer activity both in vitro and in vivo [15]. However, its precise mechanism of action against non-small cell lung cancer (NSCLC) remains incompletely understood. In this study, we investigated the effects of isorhamnetin on NSCLC cell lines A549 and H1650, with a focus on cell viability, apoptosis, cell cycle progression, as well as invasion, migration, and adhesion. Consistent with previous reports [13,14,15,16,17], isorhamnetin significantly inhibited cell viability in both A549 and H1650 cells by inducing apoptosis and causing G1/S-phase arrest. Notably, stronger inhibitory effects—including more pronounced apoptosis induction and S-phase arrest—were observed in H1650 cells compared with A549 cells, suggesting a degree of cell type-dependent activity. Furthermore, isorhamnetin markedly suppressed invasion, migration, and adhesion in both cell lines, confirming earlier findings that it can reduce metastatic potential across multiple lung cancer models [13,14,15,16,17].

To elucidate the underlying molecular mechanisms, transcriptome sequencing was performed. This analysis revealed that isorhamnetin treatment resulted in significant upregulation of two differentially expressed genes (DEGs), *ELFN1* and *TMEM186*. Meanwhile, previous studies have reported that isorhamnetin upregulates FGFBP1 protein expression while downregulating *SETDB1* protein levels. Based on these observations, we propose a multi-target synergistic model to explain the complex anticancer effects of isorhamnetin in NSCLC.

The concurrent modulation of four key proteins—*ELFN1*, *TMEM186*, *SETDB1*, and FGFBP1—highlights the polypharmacological nature of isorhamnetin, likely converging on interconnected networks that control the cell cycle, apoptosis, and metastatic potential. The coordinated action of these four proteins creates a synergistic, insurmountable stress on the cancer cell. The epigenetic and signaling shifts (*SETDB1*↓, *FGFBP1*↑) serve to disable pro-survival networks and activate checkpoint responses. Concurrently, the metabolic and adhesive stresses (*TMEM186*↑, *ELFN1*↑) generate intrinsic damage signals. These signals ultimately converge on master regulators: the DNA damage/checkpoint pathway, activating p21 to halt the cell cycle in S-phase, and the mitochondrial apoptosis pathway, tipping the Bax/Bcl-2 balance to execute cell death.

The inhibitory effect of isorhamnetin on tumor cell phenotypes is primarily manifested through its precise regulation of key modulators. SET domain bifurcated histone lysine methyltransferase 1 (*SETDB1*) is a histone methyltransferase that catalyzes the trimethylation of histone H3 at lysine 9 (H3K9me3), thereby repressing gene transcription. In various malignancies, *SETDB1* promotes tumorigenesis by silencing tumor suppressor genes (e.g., p53, p21). Conversely, depletion of *SETDB1* can induce DNA damage and senescence-like phenotypes. Our results demonstrate that isorhamnetin significantly downregulates *SETDB1* expression, a finding consistent with the work of Krossa et al., who identified *SETDB1* as essential for uveal melanoma growth and demonstrated that its loss triggers DNA damage and senescence-like responses [23]. By inhibiting *SETDB1*, isorhamnetin may relieve the repression of cell cycle inhibitors, thereby initiating the S-phase arrest observed in this study. Additionally, the downregulation of *SETDB1* might elevate replication stress by affecting the expression of DNA replication-associated genes (such as CDC6 and MCM6), ultimately culminating in apoptotic cell death [24].

Conversely, isorhamnetin markedly upregulated the expression of fibroblast growth factor binding protein 1 (FGFBP1). *FGFBP1* is a secreted protein that binds to and releases fibroblast growth factors (FGFs), thereby modulating FGF signaling activity. Within the tumor microenvironment, FGF signaling typically promotes angiogenesis, proliferation, and metastasis [25]. However, the upregulation of FGFBP1 induced by isorhamnetin may exert a complex, double-edged sword effect. According to research by Zhang Dongmei’s team, high FGFBP1 expression in colorectal cancer facilitates angiogenesis and tumor growth by activating the FGF2–FGFR1–ERK1/2–EGR1 axis to induce FAPα expression in hepatic stellate cells [26]. Therefore, isorhamnetin-induced *FGFBP1* upregulation could represent a compensatory response or act by altering the local concentration and bioavailability of FGFs, indirectly disrupting tumor cells’ dependence on growth factor signaling and consequently suppressing their migratory and invasive capacities. This disruption of the growth factor microenvironment is highly correlated with the cell adhesion inhibition phenotype observed with isorhamnetin treatment.

Transcriptomic analysis also identified two upregulated genes, *ELFN1* and *TMEM186*, providing novel perspectives on isorhamnetin’s mechanism of action. Extracellular leucine rich repeat and fibronectin type III domain containing 1 (*ELFN1*) functions primarily as a postsynaptic protein in the nervous system, but its role in oncology remains relatively underexplored. Studies have indicated that *ELFN1* is overexpressed in colon cancer and is associated with malignant progression, potentially acting through the Wnt/β-catenin signaling pathway or by influencing extracellular matrix (ECM) adhesion [27]. Moreover, long non-coding RNAs (lncRNAs) related to *ELFN1* play more prominent roles in carcinogenesis. For instance, ELFN1-AS1 is highly expressed in colorectal cancer and maintains cancer stemness by stabilizing hnRNPA1 or promotes tumorigenesis by regulating the miR-4270/AURKB axis [28]. More notably, ELFN1-AS1 forms a complex with EZH2/DNMT3a, epigenetically repressing the expression of the tumor suppressor MEIS1 and mediating chemoresistance to agents like oxaliplatin [29]. In this study, the isorhamnetin-induced upregulation of *ELFN1* is unlikely to be a simple pro-tumorigenic reaction. Instead, it may alter cell–matrix interactions, leading to aberrant adhesion (consistent with the observed anti-adhesion phenotype) or disrupt specific signaling cascades (e.g., Wnt/β-catenin) to suppress cellular behavior. Furthermore, the upregulation of *ELFN1* may be linked to the regulation of its associated lncRNAs. Isorhamnetin might perturb tumor cell metabolic reprogramming by targeting *ELFN1* and its transcripts, thereby enhancing drug sensitivity. We hypothesize that the induction of ELFN1 represents a cellular adaptive response to drug-induced stress, which ultimately contributes to the suppression of migration and invasion.

Transmembrane protein 186 (*TMEM186*) is a member of the transmembrane protein family, whose members play complex roles in cancer. While some, like *TMEM16A* and TMEM17, are oncogenic, others, such as *TMEM25* and *TMEM100,* exhibit tumor-suppressive functions [30]. The specific function of TMEM186 remains poorly characterized, although transmembrane proteins generally participate in membrane trafficking, ion channel activity, or signal transduction [31]. The isorhamnetin-induced upregulation of TMEM186 suggests a potential perturbation of the cell membrane structure or function. Such interference could lead to an imbalance in intracellular ion homeostasis or aberrant signal transduction, subsequently triggering endoplasmic reticulum (ER) stress or mitochondrial dysfunction, and ultimately driving cells towards apoptosis. In conjunction with the observed apoptotic phenotype, the upregulation of *TMEM186* may be a key mediator of isorhamnetin-induced cell death.

**Figure 11:**
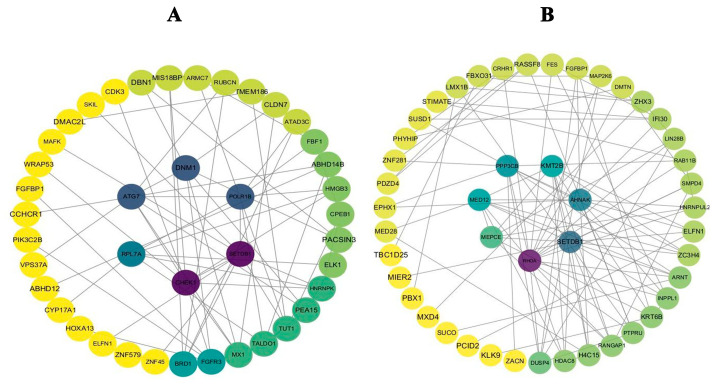
Construction of protein–protein interaction (PPI) networks in A549 and H1650 cells. (**A**) PPI network constructed from differentially expressed genes (DEGs) in A549 cells following isorhamnetin treatment. (**B**) PPI network derived from H1650 cells under the same conditions. Nodes represent proteins, with colors indicating functional modules or expression trends (yellow: downregulated; green: upregulated; blue/purple: central hubs). Edges represent known or predicted interactions based on the STRING database.

The construction of PPI networks (Figure 11) for both A549 and H1650 cells provided a systems-level perspective on how the four validated targets—SETDB1, FGFBP1, ELFN1, and *TMEM186*—interact within the cellular milieu. Intriguingly, in both cell lines, these proteins were embedded within a characteristic core–periphery network architecture. *SETDB1* and FGFBP1 consistently emerged as central hubs, suggesting their potential roles as master regulators that integrate signals from diverse pathways. In contrast, ELFN1 and TMEM186 functioned as peripheral effectors, likely executing specific downstream functions in response to these upstream hubs. Although the identity of some interacting partners varied slightly between the two cell lines (e.g., ATG7 and CHEK1 in A549 versus PPP3CB and KMT2B in H1650), the fundamental modular organization—linking epigenetic regulation, cell cycle control, and inflammatory signaling—was remarkably conserved. This cross-cell line consistency strongly argues against a cell-type-specific artifact and instead points to a shared molecular framework underlying isorhamnetin’s anti-NSCLC action. Our findings therefore support a model wherein isorhamnetin exerts its multifaceted effects not by targeting a single molecule, but by coordinately rewiring a conserved, multi-target regulatory network.

To further elaborate the multi-target regulatory network of isorhamnetin, we employed an unbiased CMap screen complemented by molecular docking simulations. This computational interrogation identified *PPARG*, *NR3C2*, and *DRD2* as high-confidence candidates whose signatures significantly converged with isorhamnetin treatment. Furthermore, molecular docking utilizing high-resolution crystal structures (PDB IDs: 6TSG, 4X9W, 6CM4) corroborated these findings, demonstrating that isorhamnetin occupies the orthosteric binding pockets of these receptors with high affinity (Vina scores: −8.3, −8.1, and −9.1 kcal/mol, respectively) (Figure 10). While our study prioritizes the mechanistic dissection of the transcriptomic signature, these computational predictions provide a valuable roadmap for future investigations.

Integrating these in silico predictions with our core findings (*SETDB1*, *FGFBP1*, *ELFN1)*, we can construct a multi-target synergistic model for isorhamnetin’s antitumor action. Isorhamnetin disrupts the epigenetic homeostasis of tumor cells by downregulating *SETDB1*, relieving the suppression of tumor suppressor genes and potentially initiating DNA damage. This provides the priming signal for cell cycle arrest and apoptosis. Simultaneously, by upregulating *FGFBP1*, it interferes with growth factor signaling within the tumor microenvironment, weakening the stroma upon which tumor cells depend for survival, thereby inhibiting proliferation, migration, and invasion. The transcriptomically identified genes *ELFN1* and *TMEM186* likely represent components of the cellular stress response machinery. Upregulated *ELFN1* may enhance anoikis sensitivity by disrupting cell-substrate interactions, while the upregulation of *TMEM186* could directly mediate dysfunction of cellular membranes or organelles, culminating in cell death. The computational nomination of *PPARG* expands the mechanistic paradigm to include metabolic reprogramming [32], synergizing with the epigenetic disruption mediated by *SETDB1*. Similarly, the identification of *DRD2* extends our understanding to the burgeoning ‘neuro-oncology’ axis in lung cancer pathobiology [33], complementing the cellular stress responses induced by *ELFN1* and *TMEM186*. Collectively, this multimodal interrogation establishes a foundational framework for elucidating isorhamnetin’s polypharmacology.

Beyond these newly identified proteins, the established pharmacological profile of flavonoids invites consideration of other potential mechanisms [34]. For instance, structural analogs like quercetin are known inhibitors of topoisomerases [35]. While our transcriptomic data did not reveal a strong DNA damage response signature (e.g., significant TP53 induction), it is plausible that isorhamnetin could interact with topoisomerases as a catalytic inhibitor rather than a poison. Such an activity might modulate DNA topology without triggering a robust transcriptional alarm [36]. This represents a plausible, yet unconfirmed, extension of its mechanism that merits future investigation.

An intriguing aspect of our data is the conjunction of S-phase arrest, potent p21 induction, high IC_50_, and the requirement for prolonged exposure. This profile is characteristic of many agents that induce therapy-induced senescence (TIS), a durable state of cell cycle arrest distinct from apoptosis [37]. Senescent cells remain metabolically active and viable, which would lead to an overestimation of cell viability in MTT assays and thus a higher apparent IC_50_—a potential explanation for the potency values observed in this study. Furthermore, the establishment of senescence involves extensive transcriptional reprogramming, consistent with the 72 h timeframe needed for isorhamnetin’s maximal effect.

### Limitations and Future Perspectives

While this study delineates the novel targets and effects of isorhamnetin, several limitations must be acknowledged to contextualize the findings and guide future research.

1. While this study elucidates the anti-tumor mechanisms of isorhamnetin in NSCLC cells, several limitations warrant attention. Although isorhamnetin exerts effects at relatively high micromolar concentrations (≤80 µM) over 72 h—a profile common for natural flavonoids—questions regarding its translational potency, bioavailability, and intracellular stability remain [38,39,40]. Crucially, although literature evidence suggests the low cytotoxicity of isorhamnetin in normal bronchial epithelial cells (BEAS-2B) within this range [41], direct cytotoxicity validation was not performed in this study. Additionally, while isorhamnetin is reported to have a more favorable safety profile than cisplatin [42,43], head-to-head comparative efficacy studies were lacking. Future investigations should employ pharmacokinetic analyses (e.g., LC-MS/MS) and include normal cell controls to precisely define the compound’s therapeutic window and confirm its potential as an adjuvant agent.
2. Mechanistic causality and interconnectivity: Although we identified changes in ELFN1, TMEM186, SETDB1,0 and FGFBP1, the study does not establish causal relationships. It is unclear whether the modulation of these proteins is a direct or indirect effect of isorhamnetin, and how their functions interrelate within a unified signaling network. The paradoxical upregulation of the putative oncogene ELFN1alongside growth inhibition underscores this complexity. Future work must employ genetic tools (CRISPR, siRNA) and protein interaction studies (Co-IP, SPR) to validate these targets as direct mediators of isorhamnetin’s efficacy and to map their pathway crosstalk (e.g., Wnt/β-catenin, PI3K/AKT) [27,44].
3. Role of cellular senescence and ROS: The induction of S-phase arrest and p21 upregulation hints at the possible involvement of therapy-induced senescence, which could explain the gradual anti-proliferative effect [45]. However, a classic senescence-associated secretory phenotype (SASP) was not evident in our transcriptomic data, which may indicate a “quiet” senescence state or a delayed SASP onset [46]. Concurrently, the potential dual role of isorhamnetin as an antioxidant or, at high concentrations, a pro-oxidant agent remains unexplored [47,48]. The contribution of oxidative stress to the observed apoptosis is unknown. Direct measurements of ROS levels and senescence markers (e.g., SA-β-gal staining) in future studies are crucial to dissect these mechanisms [49].
4. In vivo validation and clinical relevance: All conclusions are derived from in vitro models. The anti-NSCLC efficacy and mechanism of isorhamnetin, particularly the roles of the identified protein targets, require validation in animal models and, ultimately, clinical settings to assess their therapeutic relevance [12].

In conclusion, this study demonstrates that isorhamnetin exerts multi-faceted anti-NSCLC effects by concurrently targeting a network involving *ELFN1*, *TMEM186*, *SETDB1*, and *FGFBP1*. These findings not only expand the mechanistic understanding of isorhamnetin but also reveal novel potential therapeutic nodes in NSCLC. Addressing the outlined limitations through targeted mechanistic and translational studies will be essential to harness the full therapeutic potential of this natural compound.

## 5. Conclusions

This study elucidated the in vitro NSCLC cell viability and migration inhibitory effects of isorhamnetin and the related molecular mechanism involved. Isorhamnetin inhibited NSCLC cell viability in a time- and concentration-dependent manner. It also concentration-dependently induced NSCLC cell apoptosis and cell cycle arrest at the S phase. Moreover, isorhamnetin significantly inhibited the invasion, migration and adhesion rate of NSCLC cells. Furthermore, isorhamnetin upregulated *ELFN1* and *TMEM186* gene and protein expression levels, whereas it upregulated *FGFBP1* protein expression levels and downregulated SETDB1 protein expression levels in NSCLC cells. To sum up, isorhamnetin may inhibit NSCLC cell viability and migration via the upregulation of *FGFBP1*, *ELFN1* and *TMEM186* protein expression levels and via the downregulation of SETDB1 protein expression levels in NSCLC cells.

## Figures and Tables

**Table 1:** Primer sequences for qRT-PCR.

Gene Name	Forward (5′ to 3′)	Reverse (5′ to 3′)
*TMEM186*	TACGGAGACTGGTTGGTACCT	GCTGGATACGCACAAACACTC
*ELFN1*	CTCAACCTCACCAAGAACGAGA	CAGGTCGATGTTGACGATGTTG
*GAPDH*	CATGAGAAGTATGACAACAGCCT	AGTCCTTCCACGATACCAAAGT

## Data Availability

The datasets generated and/or analyzed during the current study are included in the article and its Supplementary Materials files.

## Supplementary Materials

The following supporting information can be downloaded at https://www.mdpi.com/article/10.3390/biomedicines14050951/s1, Figures S1 and S2. Induction of apoptosis in A549 (A) and H1650 (B) cells by the compound (Annexin V/PI double staining flow cytometry). Groups include the control group (CON) and compound treatment groups (0.1, 1, 10, 100, and 200 μM). The four quadrants represent viable cells (Annexin V^−^/PI^−^), early apoptotic cells (Annexin V^+^/PI^−^), late apoptotic/necrotic cells (Annexin V^+^/PI^+^), and damaged cells (Annexin V^−^/PI^+^), respectively. The vertical axis indicates the percentage of apoptotic cells (error bars: SD). Table S1. RNA-seq analysis (differentially expressed genes in isorhamnetin treated A549 cells). Table S2. RNA-seq analysis (significantly differential expressed genes in isorhamnetin treated A549 cells). Table S3. Significantly differential genes in isorhamnetin treated A549 cells (*p* < 0.05, Log 2FC > |2|). Table S4. GO analysis (A549). Table S5. KEGG analysis(A549). Table S6. RNA-seq analysis (differentially expressed genes in isorhamnetin treated H1650 cells). Table S7. RNA-seq analysis (significantly differential expressed genes in isorhamnetin treated H1650 cells). Table S8. significantly differential genes in isorhamnetin treated H1650 cells (*p* < 0.05, Log 2FC > |2|). Table S9. GO analysis (H1650 cell). Table S10. KEGG analysis (H1650 cell).
