# Supplementary figures and images for: In Vitro Cell Viability and Migration Inhibitory Effects of Isorhamnetin in Non-Small Cell Lung Cancer Cells
Source: Biomedicines. 2026 Apr 22;14(5):951. doi: 10.3390/biomedicines14050951 (PMC13205021; doi:10.3390/biomedicines14050951)

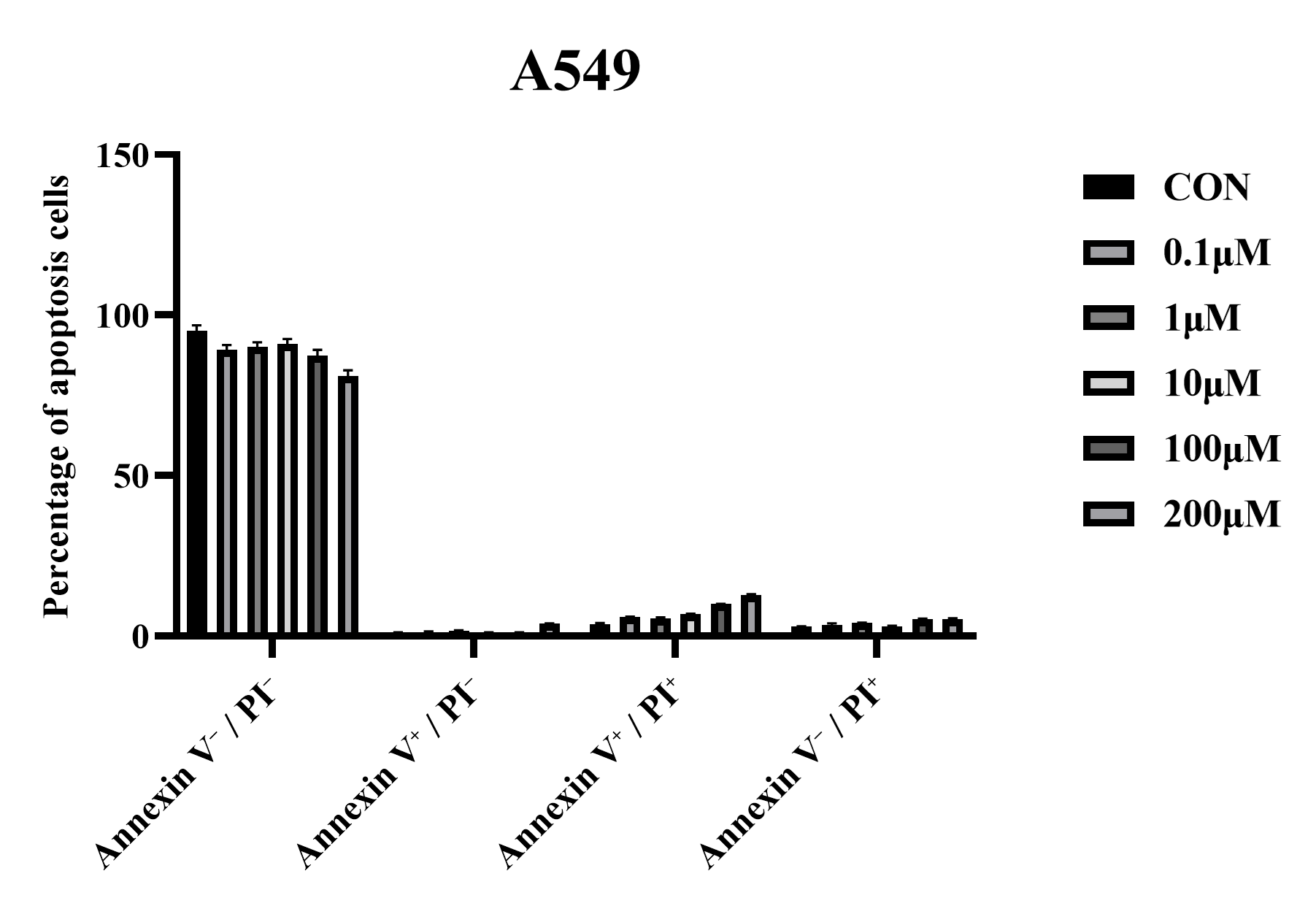

Supplement: Supplementary file 1 [file biomedicines-14-00951-s001.zip › Figure S1.png]

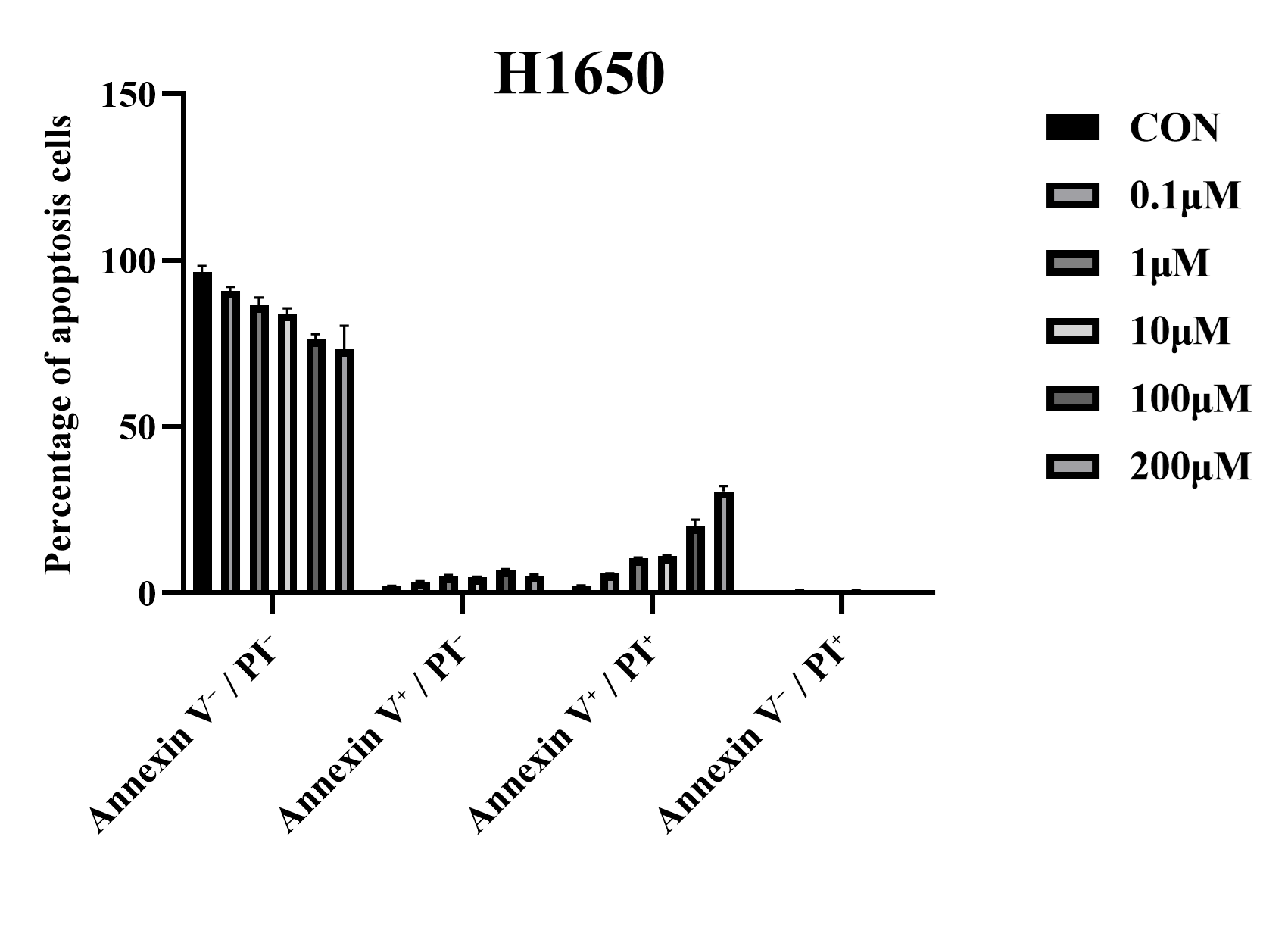

Supplement: Supplementary file 1 [file biomedicines-14-00951-s001.zip › Figure S2.png]
